# Sexual and Reproductive Health Problems of Female University Students in Iran: A Qualitative Study

**DOI:** 10.5539/gjhs.v7n4p278

**Published:** 2015-01-14

**Authors:** Fatemeh Yari, Zahra B. Moghadam, Soror Parvizi, Nahid D. Nayeri, Elham Rezaei

**Affiliations:** 1 Department of Reproductive Health, Nursing and Midwifery Faculty, Tehran University of Medical Sciences, Tehran, Iran; 2 Department of Pediatric Nursing, Nursing and Midwifery Faculty, Center for Educational Research in Medical Sciences (CERMS), Iran University of Medical Sciences, Tehran, Iran; 3 Nursing and Midwifery Care Research Center, School of Nursing and Midwifery, Tehran University of Medical Sciences, Tehran, Iran; 4 Department of Midwifery, Nursing and Midwifery Faculty, Urmia University of Medical Sciences, Urmia, Iran

**Keywords:** young, reproductive health, qualitative study

## Abstract

**Objective:**

Youth is defined as the time of transition into adulthood and an important period in a person’s life. During this period new behavior is learned easier than adulthood. Therefore, special attention has to be necessarily paid to this period in order to promote the health. Addressing adolescent reproductive health issues is also a critical factor

**Methods:**

This research was a qualitative study conducted from January 2014 to July 2014. Data from focus group discussions and semi-structured interviews with 25 female students and 10 key members of the university (including university authorities, consultants, reproductive health professionals and university officials) was collected and all interviews were recorded, formulated and classified.

**Results:**

The mean age of participants was 22.43 years. A total of 8 students majored in geology, 5 majored in chemistry, 3 in statistics, 3 in mathematics, and 6 in biology. 17 had a bachelor’s degree, 3 master’s degree and 5 doctorate degree. Majority of students (82.4%) were never married and 23 of them lived in dormitories. The following three main themes were extracted from the interviews: Reproduction thought as pregnancy; the taboo of sex; and inappropriate relation between parents and children.

**Conclusion:**

Most participants stressed the need to provide reproductive health services for young girls.

## 1. Introduction

Youth is defined as the time of transition into adulthood and an important period in a person’s life. During this period new behavior is learned easier than in adulthood (UNICEF, 1997; MOH, 2006). This fact was stressed in the International Conference on Population and Development in 1994 (Hagikhani et al., 2012). In this regard, the World Health Organization (WHO) estimates that more than one billion people in the world are between 15-24 years old (UNICEF, 1997; WHO, 2008). Moreover, more than 85% of them live in developing countries. Investment in the health of this age group has played a major role in the development of human communities due to the dual role of women in community health and well-being of future generations as one of the main paths to the achievement of Millennium Development Goals (MDGs) and youth goals (Parvizi et al., 2011).

In connection with the discussion of health, sexual and reproductive health is an important part of world health (UNICEF, 1997; Li Ping, 2012) and as a part of human rights it has been approved for public (Mazloomy, 2007). In this regard, many young women have very little reproductive health information (Li Ping, 2012). In addition, it has been reported that the young are facing different sexual and reproductive health problems like unwanted pregnancy, unsafe abortion, and STI including HIV (WHO, 2005). It is estimated that many women in the world die due to complications during pregnancy and abortion every year. On the other hand, the mortality rate caused by unwanted pregnancy and sexually transmitted diseases (STD) is increasing around the globe (WHO, 2008).

Despite the concerns announced by the United Nations, 180 member countries, international organizations, and individual adolescents everywhere, the reproductive health concerns of young people are too often neglected (Greene, 2002). Most of the population in our country (i.e. more than half of the population) is under 25 years of age. According to the Statistical Center of Iran, a total of 60% of the population is under 25 years and over 50% are under 20 years (http://www.space.ir/barnameh/Baranmeh%20gozashe, 2012). Therefore, neglecting this population and their future reproductive health will bring about irreparable consequences for the adults (Bott, 2003). Hence, it is necessary to maintain the functionality of the present programs and assess emerging needs and current trends associated with this issue (Sadeghipour et al., 2006).

Based on results of the Iranian national Census, of the almost 70-millionpopulation of Iran, about 11.5 million are girls aging between 10–24 years (Hagikhani et al., 2012). As a result of concerns for this important issue, the United Nations has changed the theme of 2013 World Population Day was teenage pregnancy. This highlighted the significant of the role played by teen girls in positively influencing future generations and stressed the importance of providing them with adequate health care and educational support (Kaiser, 2013).

In Iran, girls’ health status is often considered to be more important than boys. There are several cultural and social reasons for this point of view. First, a girl’s lifespan includes several stages and milestones including infancy, childhood, puberty, marriage, pregnancy, childbearing and menopause (Hagikhani et al., 2012; Mirzaei & Olfati, 2014). Despite this trend, little is known about the reproductive health needs of young people in Iran. In addition, the few studies conducted on the knowledge, attitude, beliefs and behavior associated with sexual reproductive health of Iranian youth have indicated that the level of knowledge of reproductive health is low (Mohammadi et al., 2006; Tavakol, 2003). It is the responsibility of health researchers to identify the needs for reproductive health promotion and to plan and implement the necessary educational programs that might include prevention of STIs/HIV/AIDS as well as unwanted pregnancies (Ramezani, 2000).

In Islamic Republic of Iran, students forma large part of the population were selected to study the reproductive health status of Iranians (WHO, 2004) and students following separation from parents, which exposes them to sexual reproductive health problems like the following: love relationship, physical health problems, alcohol use, weight change, eating problems, AIDS, drug abuse, time management, fear of examinations and serious psychological concerns. Some of these concerns include depression, sadness, mood changes, anxieties and phobias (Mohammadi et al., 2006). University students in general are the hopes of every country and, unfortunately, there are no comprehensive studies in this field. The existing studies and articles are also limited to quantitative studies of youth. Most experts believe that the range of quantitative methods for the assessment of real needs sweeping capabilities is very limited and qualitative research methods are required to make it good enough (Streubert & Carpenter, 2003). Therefore, research on youth reproductive health status and requirements of Reproductive Health in Iran is important for various reasons (Vakilian et al., 2011). Since college students and alumni form important communities, these communities were selected to explain the reason. The reproductive health status of female students of Lorestan University residing in Khoramabad city (in the southwest of IRAN) was also examined. Khoramabad is the capital of Lorestan province and 22^nd^ most populated city of Iran. This city is located in the valleys of the Zagros Mountain Range at an altitude of 1147.8 meters. It accommodates a population of about 687167 people with 33% of the population being the youth (Jump, 2011). Therefore; this study was aimed to systematically explore the sexual and reproductive health problems among female students attending the University of Lorestan.

## 2. Methods

Lorestan University is the oldest higher education institute in Khoramabad. It was established in 1977as the Education Center of Lorestan. Currently, this university has 7 faculties and serves 8239 students. The present study was conducted in the faculty of sciences serving1785 students studying for bachelor’s, master’s and doctorate degrees (Lu.ac.ir, 2012). A qualitative design based on the content analysis approach was used to conduct this study aim from January 2014 to July 2014. Content analysis is a systematic method of classification and coding using which information can be extracted from text. It is used for revealing a pattern of words, repetition, and structure. It is used to analyze relationship and interactions between words, repetition and structure (Grbich, 2013; Behboodi et al., 2013).

Therefore, in this study implicit and explicit concepts were identified based on the explanations of the participants and principles of coding, summarization, categories, and themes were identified. Codes were based on meaning extracted from the participants’ descriptions. The meanings were later classified based on the discovered differences or similarities (Behboodi et al., 2013; Graneheim & Lundman, 2004).

The study population for this study included female students of Lorestan University. Data were collected through semi-structured interviews. In-depth interviews were conducted by a trained interviewer. Each interview was carried out with 25 female students who were in Khoramabad, Iran. All female students were interviewed in a private room, and each interview lasted 45-90 minutes in average. Two focus group discussions involving eight participants were conducted in two sessions. The sessions were attended by students, Reproductive Health Professionals, consultants and university authorities. The duration of focus group discussion was between 1 and 3 hours. The contents were discussed; the materials were discussed before the arrival of the participants.

The interviews questions were focused on the following themes:

- Explanation of reproductive health
- Explanation of reproductive Health issues
- Elaboration on reproductive health problems

Each interview was recorded and transcribed verbatim and then the resulting material was analyzed. Point of idea saturation was considered to end in-depth interviews and focus discussions. Concepts were merged based on their themes and a manual thematic framework analysis was also used. The results were summarized and presented in narrative forms.

**Rigor**

In this study, various aspects of trustworthiness have been observed. Credibility was established through member checking, peer checking, and prolonged engagement. Member checking was done by asking the respondents to approve the transcripts and emerging codes from the interviews. Research teams consulted with each other to deal with any ambiguities in the coding process, categories and themes researchers independently analyzed the data by identifying and categorizing codes for the subjects’ responses to each question, and then the two authors’ codes and their latest analysis development as themes were compared. In areas where the two did not agree, definitions were clarified and discussion continued until consensus was reached. For addressing transferability, the complete set of data analysis documents are on file and available upon request.

This study is a part of the first author’s doctoral dissertation. The Ethics Committee of Tehran University of Medical Sciences approved the study proposal and corroborated its ethical considerations. All participants were informed about the purposes and the methods employed in this study. The permission for typing and recording the interviews was obtained from the participants. They were informed that participation in the study was voluntary and they could refuse to participate at any time without being deprived of the services delivered to them.

## 3. Results

The mean age of participants was 22.43 years. A total of 8 students majored in geology, 5 majored in chemistry, 3 in statistics, 3 in mathematics, and 6 in biology.17 had a bachelor’s degree, 3 master’s degree and 5 doctorate degree. Majority of students (82.4%) were never married and 23 of them lived in dormitories.

The following three main themes were extracted from the interviews: Reproduction thought as pregnancy; the taboo of sex, inappropriate relation between parents and children.

**Reproduction Thought as Pregnancy**

The majority of participants mentioned reproduction thought as pregnancy. This theme had three sub themes including the following: Lack of awareness and accurate information on reproductive health; lack of coverage of sexual and reproductive health problems by the media; lack of non-medical reproductive health-related books.

**Lack of Awareness and Accurate Information on Reproductive Health**

One of the things that most participants mentioned was the lack of knowledge and information about reproductive health. Almost all students defined reproductive health in association with pregnancy. One of the participants described her experience as follows:

*“I think that reproductive health means pregnancy and the term reproductive health refers to health. In general, my knowledge of this area is very little”. (22-year-old girl)*

The primary reason for such inadequate understanding of sexual and reproductive health could be the lack of awareness increasing courses in universities.

*“We know nothing about pregnancy, contraception methods and sexual disorders”. (a 20-year-old girl)*

During the focus group discussions few of the participants stated that they did not clearly know what sexual and reproductive health was about.

Also some of the participants referred to a lack of knowledge on sexual and reproductive health issues especially among students coming from rural parts of the country.

Most students referred to the lack of centers for sexual and reproductive health care in universities.

**Lack of Coverage of Sexual and Reproductive Health Issues by the Media**

Many of the students were complaining about the lack of coverage of sexual and reproductive health issues by the media.

Most participants stated that the role of the media in the field of sexual and reproductive health was very insignificant.

One of the participants said:

*“The media has a significant impaction the education of youth on sexual and reproductive health issues. Media should provide accurate information on sexual and reproductive issues to the youth”. (a 33-year-old Reproductive Health Professional)*

Most students participated in this study believed that reproductive health information obtained through friends is not reliable and cannot be considered are liable source. Therefore, the media can eliminate these sources of non-academic information through proper training.

One of the participants said:

*“In our current community there are not many sources of information for young people. Therefore, there are few advertisers and advertisement takes place through the mass media in this regard”. (23-year-old girl)*

**Lack of Non-Medical Reproductive Health Textbooks**

Nearly all of the participants wanted sexual health education to be offered formally in their curriculum as a formal course similar to other courses. They were interested in taking a formal course on reproductive health in the university.

One of the students said:

*“We do not have non-medical books on reproductive health care…I wish we had a specific book for such things”. (a 19-year-old girl)*

**The Taboo of Sex**

This theme included the following one sub themes: cultural and social environment.

**Cultural and Social Environment**

Most participants in this study suggested that cultural barriers are a crucial factor contributing to the concealment of sexual and reproductive health problems by students. Lack of positive interaction between mothers and daughters for various reasons such as embarrassment, negative attitudes, and fear of shame, and lack of adequate information on these issues regard were among other barriers.

One of the participants described her experience as follows:

*“I’d like to get more information on reproduction, sexual transmitted diseases and risky sexual behavior, etc. We are not comfortable asking about these things at home. So I usually get my information from my friends, the Internet, magazines or books”. (a 22-year-old girl)*

Another participant stated:

*“We are not comfortable in the society and if we have a question about sex, we do not know where to go. So our knowledge of sex is poor”. (A 25-year-old girl)*

Due to cultural and social taboos set by the society, many signs of puberty in girls and their sexual development are not embraced.

One of the students stated:

*“I’m really asking questions about sex from my mother. But I am not simply comfortable asking such questions.” (20-year-old girl)*

And another participant said:

*“My mother believed these things should not be taught to girls until marriage because girls may become rude”. (18-year-old girl)*

**Inappropriate Relation Between Parent and Children**

The majority of students believed that family is a very important factor in the development of sexual behavior. This theme included the following three sub themes: ensured independence of children, lack of child control, and too much reliance on children.

One participant stated:

*“In some cultures, parents are the influential sources of knowledge, beliefs, attitudes, and values for children and the youth. If young people do not get information from their families, they will seek answers elsewhere and try to find answers through peers, the media or their observations of adult actions.”*

One of the reasons stated by the majority of students was as follows:

*“The freedom given by some families transforming from traditional to modern families to the youth is increasing and is diminishing the role of parents.”*

## 4. Discussion

Research results indicated that students of the Lorestan University have large sexual and reproductive health needs. According to the results, students’ awareness of reproductive health issues such as sexually transmitted diseases (e.g. HIV) and contraceptive and sexual health is inadequate. The lack of knowledge about sex and reproduction among youth is also a factor reported by studies carried out in many developing countries (Regmi et al., 2010; Rani & Lule, 2004).

The results of the present study regarding the most important problem with sexual and reproductive health of the youth were consistent with the results of other studies in other developing countries(Yazdi et al., 2006; Tavoosi 2004, Kamau, 2006; Munthali et al., 2013). In this regard, it is essential to enhance the knowledge of the youth and increase the participation of experts and experienced teachers in developing educational materials that comply with the cultural and religious values of society. Accordingly, young people’s hall benefit from sexual and reproductive health education and should not be deprived of this right.

The most important finding of this study was the significance of the lack of coverage of sexual and reproductive health issues by the media, as the media plays an important role in raising awareness about sexual and reproductive health (Fatch et al., 2013). The study by Kalembo al. (2013) revealed that the media plays an important role in enhancing their reproductive health of adolescents in sub-Saharan Africa. Lack of non-medical reproductive health-related books was another problem referred to by the students. Based on the young student’ opinions, not only the existing textbooks fail to provide comprehensive sexual and reproductive health information, but also there is no access to any appropriate book teaching sexual and reproductive health materials. By the Statement issued by the International Conference on Population and Developmentin 1994, it is necessary to provide access to reproductive health information and government services to the youth (UN, 1994; Broundtland, 1999). Sexual issues and lack of proper information in this regard were among the most important concerns reported by most students. The majority of the student wanted to know more about topics such as STDs and high risk sexual behaviors, which are considered to be taboos in Iran due to the cultural and social environment governing the country. A qualitative study in Iran concluded that considering the Iranian cultural and religious background, families and religious beliefs play an important role in reducing high-risk sexual behaviors among Iranian adolescents (Parvizi et al., 2005). Most students in this study reported that community does not influence sexual issues.

One of the most important experiences reported by students in this study was the modesty shown about the sexual reproductive health issues. The youth are ashamed to ask for information from adults who reluctant to discuss these issues. Hence, taboos, beliefs and traditions may prevent the youth from accessing the necessary information. In many cultures, parents do not talk about sex with their children and therefore they are useful sources of information (UNFPA, 2008). Research revealed that with an increase in the parent-adolescent communication and conversation the level of sexual risk decreases.

The present study showed that the relationship between parents and children is very poor.

Since the Iranian society is transforming from a traditional society to a modern one, many families have lost their traditional attitudes and thus children are given more independence. Research revealed that with an increase in the parent-adolescent communication and conversation the level of sexual risk decreases (Diorio et al., 1999; Kotchick et al., 1999). Other studies showed that parental control is also another factor in predicting risky sexual behavior in adolescents (Li et al., 2000). Another study reported that a warm relationship between parents and children as well as parent control can considerable contribute to the prevention of behavior risk-taking in female adolescent (Khosravi et al., 2007).

## 5. Conclusion

This study showed that the knowledge of students about sexual and reproductive health is very low and that the society and family do not have a significant role in addressing the reproductive health problems of the youth.

The following recommendations are thus provided based on the research findings:

- University authorities should provide opportunities for students to address many reproductive health issues and problems.
- Universities should increase its number of counselors to solve many of the sexual and reproductive problems faced by students.
- University authorities are strongly recommended to consider provision of friendly health services.
- There should also be a charter for sexual and reproductive health rights of female students in universities.
